# A homozygous *PRKN*-associated juvenile Parkinson's disease with pregnancy in China

**DOI:** 10.3389/fneur.2023.1103164

**Published:** 2023-02-20

**Authors:** Hong-xing Li, Mei Dong, Xiao-xiao Peng, Yi-zhe Liu, Han Wang, Chao Li, Yi-yi Du, Kai Zhang, Qiang Zong

**Affiliations:** ^1^Department of Neurosurgery, Shengli Oilfield Central Hospital, Dongying, Shandong, China; ^2^Department of Stomatology, Dongying District People's Hospital, Dongying, Shandong, China

**Keywords:** Parkinson's disease, young-onset PD, pregnancy, *PRKN* gene, levodopa/benserazide

## Abstract

**Background:**

Although Parkinson's disease (PD) is the second most common neurodegenerative disorder, pregnancy in patients with PD is a relatively rare occurrence because the most common age of onset of PD is beyond the childbearing age, except in patients with Young-Onset PD (YOPD) caused by parkin RBR E3 ubiquitin protein ligase (*PRKN*) mutations.

**Case:**

In this study, we report the case of a 30-year-old Chinese woman who was affected by *PRKN*-associated YOPD and was treated with levodopa/benserazide during pregnancy. She gave birth to a healthy baby boy with an Apgar score of 9 through an uncomplicated vaginal delivery.

**Conclusion:**

This case further suggests that levodopa/benserazide during pregnancy is safe in the treatment of *PRKN*-associated YOPD.

## Introduction

Parkinson's disease (PD), first described by James Parkinson in 1817, is the second most common neurodegenerative disease after Alzheimer's disease, caused by the degeneration of dopaminergic neurons in the substantia nigra pars compacta of the midbrain (1). The main motor symptoms of PD include resting tremors, bradykinesia, rigidity, and postural instability, as well as a wide range of non-motor symptoms such as autonomic, sensory, sleep, and neuropsychiatric dysfunctions (2). PD is a geriatric disease affecting more than 1% of individuals aged 55 years and more than 3% of individuals aged 75 years and over, with the average age of the onset of PD being 60 years (3, 4). Only ~5% of patients diagnosed with PD are below 40 years of age (3–6).

The etiology of PD, which is heterogeneous, multifactorial, and often complex, is as elusive as it was first described in 1817. Recent epidemiologic studies from around the world indicated that genetic risk factors are involved in the pathogenesis of PD. Genetic studies reported that mutations in α-synuclein (SNCA; *PARK1*) and leucine-rich repeat kinase 2 (LRRK2; *PARK8*) result in autosomal dominant PD, and mutations in parkin RBR E3 ubiquitin protein ligase [*PRKN*; parkin (*PARK2*)], DJ-1 [Parkinson protein 7 (*PARK7*)], and PTEN-induced putative kinase 1 (PINK1; *PARK6*) result in autosomal recessive PD (4, 7–9). *PRKN* gene (*PARK2*) mutation was initially reported in a sample of Japanese families with juvenile parkinsonism and was the most common cause of autosomal recessive PD, which was located on Chromosome 6 and was particularly prevalent in women with Young-Onset PD (YOPD), with the onset of PD before the age of 30 years (7, 8, 10). Therefore, there is a possibility that after diagnosis with *PRKN*-associated YOPD, a woman could become pregnant (3–5). However, in all cases of PD, mutations in these genes may result in fewer than 5% of cases (7). Pregnancy in patients with PD may be an uncommon occurrence (6), and in patients with *PRKN*-associated YOPD, it is even more uncommon. The question of how to manage these two situations is critical to the health of both mothers and children. However, information on the clinical experience of pregnancy management in patients with PD is limited to case reports only. To our knowledge, there has never been a report demonstrating the effect of autosomal recessive *PRKN* mutations on pregnancy in Chinese individuals with YOPD. In the present study, we describe the case of a Chinese woman with YOPD associated with a homozygous mutation of the *PRKN* gene treated with low-dose levodopa/benserazide during her pregnancy. Through this case, we hope to guide neurologists and movement disorder experts to understand pregnancies in patients with PD better to ensure the best treatment for both mothers and children.

## Case report

We report the case of a 36-year-old Chinese woman with an 18-year history of PD. The extrapyramidal diseases were negative in her family history, but her parents had a consanguineous marriage (Her mother's grandmother and her father's great-grandfather were shared parents). There was no history of drug, alcohol or tobacco consumption, poisoning, or head injury. Her symptoms started at the age of 18 years with a slow progression, and resting tremors, bradykinesia, and rigidity developed later. Her initial symptom was dystonia of the left lower limb, which was characterized by the toes being stiff and flexed during walking. She was diagnosed with dopamine-responsive dystonia at the age of 24 years and given levodopa/benserazide (50/12.5 mg/day) treatment, and consequently, she obtained considerable therapeutic benefit. Her symptoms improved significantly and progressed slowly, she did not return to the department for further consultation, and she self-adjusted her medications according to her symptoms.

At the age of 30 years, she was confirmed as being pregnant and referred to our department. Her motor symptoms were well-controlled by taking levodopa/benserazide 100/25 mg two times a day. The neurological examination revealed clear symptoms of PD (“off” periods), including mild hypomimia, mild rigidity of the bilateral limbs, bradykinesia, resting tremor (predominant on the left), and dystonic posture of her left leg. Other neurological examinations were normal. Her total score on the Unified Parkinson's Disease Rating Scale motor section (UPDRS III) was 16, and the Hoehn and Yahr (H-Y) staging was II. Her laboratory results (including routine blood examination, ceruloplasmin, and thyroid-related hormones) and the brain magnetic resonance imaging (MRI) findings were normal. According to the Movement Disorder Society's clinical diagnostic criteria for PD, the patient was diagnosed with PD.

During the first trimester of her pregnancy, her PD symptoms were similar to the preconception period. However, in gestation week 16, the patient refused to continue taking levodopa/benserazide because her PD symptoms improved for no apparent reason, with the UPDRS III being 6. At 32 gestational weeks, she gave birth to a healthy baby boy with an Apgar score of 9 through an uneventful vaginal delivery without complications. The infant was fed milk formula to avoid exposure to antiparkinsonian (anti-PD) drugs. Two weeks after her pregnancy, the PD symptoms of the patient became aggravated; therefore, she continued to take levodopa/benserazide (100/25 mg two times a day) and achieved a good curative effect. The child was followed up for 6 years, and his general neurological examination and the routine blood tests were normal.

**Figure 1:**
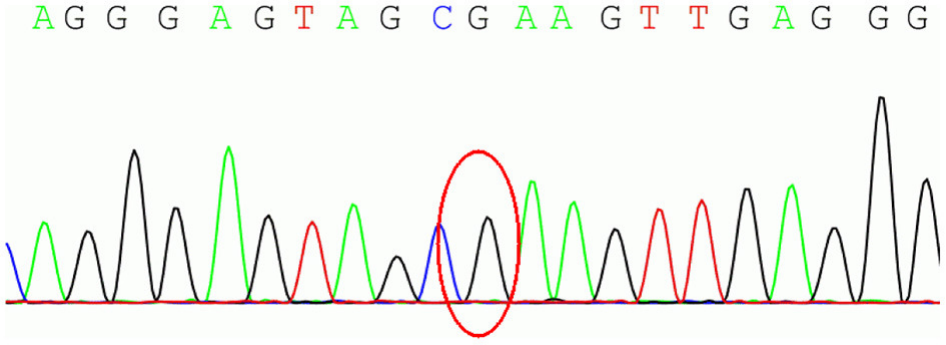
The homozygous mutation of p.G284R (chr6-162,206,825, c.850G > C) in exon 7 of parkin RBR E3 ubiquitin protein ligase (*PRKN*).

**Figure 2:**
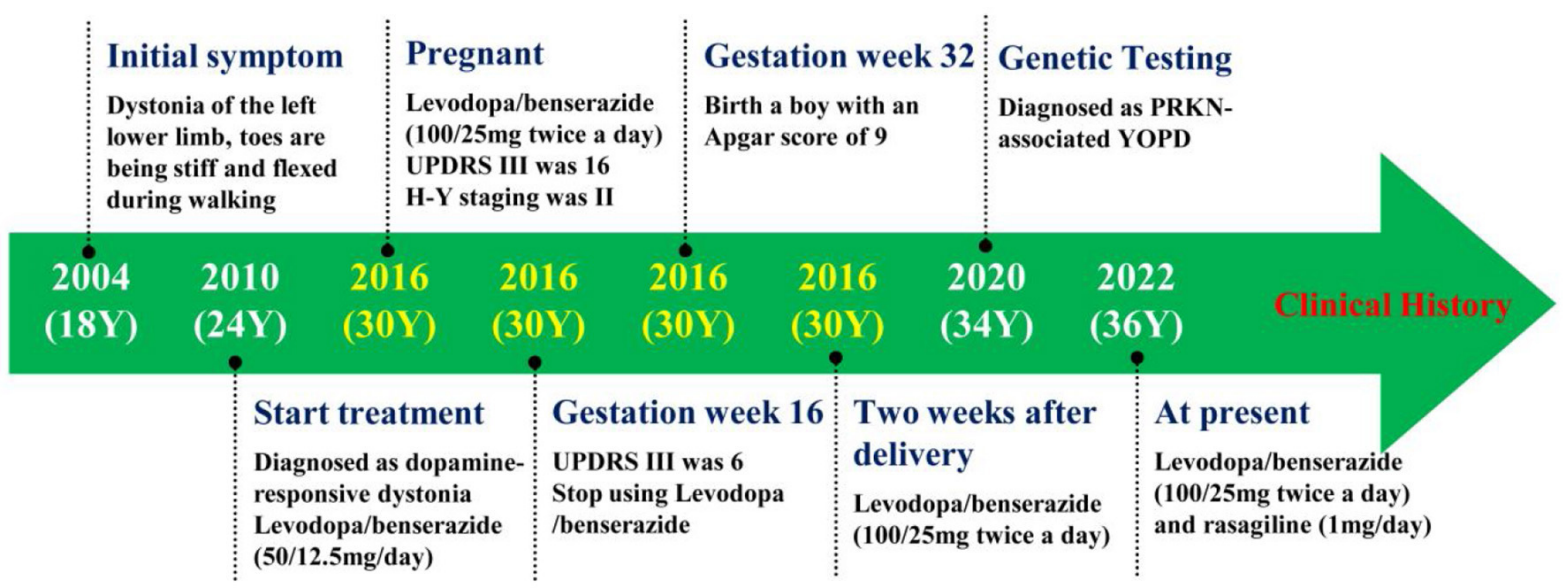
The figure showing the patient's clinical history. Y, years old; UPDRS III, Unified Parkinson's Disease Rating Scale motor section; H-Y, Hoehn and Yahr; YOPD, Young-Onset Parkinson's Disease.

To further define the diagnosis and identify the causative variant, at the age of 34 years, we recommended complete exome sequencing monitoring of the patient and her parents. Genomic DNA was extracted from the peripheral blood leukocytes using standard procedures. High-throughput sequencing and exon capture technology were performed. A homozygous mutation of p.G284R (chr6-162,206,825, c.850G > C) in exon 7 of *PRKN*, which was inherited from her unaffected father and mother, was detected. The sequencing results are shown in Figure 1. This mutation was demonstrated to be a pathogenic mutation for PD. All these results confirmed the diagnosis of autosomal recessive YOPD due to the *PRKN* homozygous mutation. At present (age 36 years), the patient is taking rasagiline 1 mg/day, in addition to levodopa/benserazide 100/25 mg two times a day, and her neurological status is currently stable. To show the patient's onset and treatment process more intuitively, an illustration of this patients clinical history has been drawn and is shown in Figure 2.

## Discussion

The incidence of pregnancy during PD is unknown. PD is a disease that mostly affects older individuals. Thus, pregnancy in PD is uncommon, especially in patients with YOPD caused by *PRKN* mutations. In this study, we report for the first time a Chinese woman who was diagnosed with sporadic YOPD associated with a compound heterozygous mutation of the *PRKN* gene. She was treated with low-dose levodopa/benserazide during pregnancy and later gave birth vaginally to a healthy baby boy.

The etiology of PD is heterogeneous, multifactorial, and usually complex. For many years, PD has been considered to be caused by environmental factors. However, growing research shows that genetic factors seem to play a role in at least a subset of PD patients (7). *PRKN* gene (*PARK2*) mutations are the most common cause of autosomal recessive PD, especially prevalent in patients with PD, with onset before the age of 30 years. The gene located on Chromosome 6 was first identified in a sample of Japanese families with juvenile Parkinsonism (8, 10). To date, more and more allele variants of the gene, including point mutation and exon rearrangements (such as deletion or duplication), have been found, which complicate Parkinson genotyping (10–12). In 2000, Lucking et al. performed a study to analyze 73 families and found that mutations in the *parkin* gene were detected in 36 out of 73 families. This study also indicated that *parkin* gene mutation was the main cause of early onset of autosomal recessive familial PD and isolated juvenile-onset PD (≤ 20 years old) (13). Since the first gene of PD was discovered 25 years ago (10, 14), more and more PD-related genes have been discovered. Therefore, in the clinical study, we recommend that genetic testing should be carried out for YOPD, hereditary or abnormal parkinsonian disorders, to perform a clear diagnosis.

At present, the pathogenesis of the *PRKN* gene lacks clarity. The *PRKN* gene could encode the Parkin protein. It is an E3 ubiquitin ligase protein that plays an important role in the normal functioning of the mitochondria and the ubiquitin–proteasome system. It can catalyze the transfer of ubiquitin to its specific target protein, guide protein degradation in the proteasome, and prevent cell apoptosis. The mutation of *PRKN* leads to a loss or decrease in the function of Parkin protein and destroys the activity of E3, thus increasing the risk of PD (7, 8).

The phenotypes of *PRKN* mutation are as follows: (1) The patient's age at the onset of the disease is early, mostly around 30 years. (2) It has typical PD features. (3) Dystonia is often symmetrical and more common. (4) Disease progression is slow. (5) An excellent response with levodopa was noted, but with frequent complications (levodopa-induced dyskinesia and fluctuation). (6) Cognitive impairment is rare (7, 8). In this case, the age of the onset of PD symptoms was 18 years, and the initial symptom was dystonia of the left lower limb. She responded excellently to levodopa and made slow progress. At present, although she has been suffering from this disease for the past 18 years, she only requires a low-dose of levodopa/benserazide to maintain a normal life without any cognitive impairment. It is consistent with the theory that the *PRKN* gene causes autosomal recessive juvenile PD.

The literature analyzing the effect of pregnancy on the motor symptoms of PD is controversial (6). Some studies indicated that pregnancy worsens the clinical symptoms of patients (15–17), but other reports showed that patients' symptoms remained stable (17, 18) or that the patients even displayed improvement in PD symptoms throughout (8, 19). The physiological mechanism of pregnancy leading to a change in symptoms is still complex. Several theories on worsening symptoms during pregnancy were proposed, including (1) the natural progression of PD; (2) the patient's plasma volume, the volume of distribution, diet, intestinal absorption, and metabolic state being altered during pregnancy (20); (3) the changes in physiological and psychological stress during pregnancy; and (4) changes in estrogen levels (6). The symptoms experienced by the patient improved during pregnancy, and she even stopped levodopa/benserazide. This improvement in the patient's symptoms may be related to an increase in the estrogen level in her body during pregnancy. Animal models suggest that estrogen has a neuroprotective effect on the dopaminergic neurons (21). Several epidemiologic studies suggest that estrogen has a protective effect on PD (6, 21–23). However, several population studies suggest that there is no link between estrogen exposure and the risk of PD (24, 25). Some studies even suggest that estrogen exposure increases the risk of PD (26). Therefore, the role of estrogen in the risk of PD is ambiguous.

Experience in the use and safety of anti-PD medication during pregnancy in patients with PD is minimal and limited to case reports and small case series. The main focus of the management of pregnancy in patients with PD is the safety of anti-PD medication on the fetus. Owing to the lack of human or animal evidence on their effects on fetal development, all anti-PD medications are contraindicated during pregnancy (27, 28). Undoubtedly, levodopa is the most effective drug for PD, and it also has the most widespread use and acceptance during pregnancy. Many studies used levodopa alone or in combination with dopa-decarboxylase inhibitors (carbidopa or benserazide) to treat PD during pregnancy. Studies showed that levodopa can cross the placenta and be metabolized by the fetus, but carbidopa and pheniramine do not cross the placenta or enter fetal circulation (29). Although some animal studies demonstrated that the levodopa treatment does not affect the pregnancy or the fetus, during levodopa monotherapy or dopamine decarboxylase therapy, however, two cases of early pregnancy miscarriage (30, 31) and one case of fetal osteomalacia (32) were reported. However, two reports published earlier indicated that no teratogenic effect was reported in seven cases of levodopa used as monotherapy (33, 34). In Mara Seier's review from 2017, who analyzed 114 pregnancies (47 women with PD and 67 other disorders) exposed to levodopa, the results showed that levodopa did not increase the rate of miscarriage, birth complications, or teratogenicity during pregnancy (6). In our case, the patient continued to take levodopa/benserazide during pregnancy, and her symptoms remained stable or even improved. Although the baby was born through premature delivery, the reason for this premature delivery was considered to be due to cervical incompetence. Finally, the woman and the baby did not have any complications, which further proved that levodopa combined with dopamine carboxylase was relatively safe.

Although ergot-derived dopamine agonists (cabergoline, bromocriptine, and diuretic) have been used for the treatment of infertility in women with hyperprolactinemia for decades, there is little evidence to suggest that dopamine agonists are used in pregnancy to treat women with PD. Seier et al. (6) summarized 14 cases of pregnancy of individuals with PD who were exposed to dopamine agonists: three were exposed to pramipexole, three were exposed to ropinirole, five were exposed to bromocriptine, two were exposed to cabergoline, and one was exposed to pergolide. In these 14 cases, no teratogenicity was reported but one baby had a seizure shortly after birth but with subsequent normal development, and one placental abruption did occur (6). Based on these data, it is not enough to recommend the routine use of dopamine agonists during pregnancy. In animal and human studies, amantadine is associated with teratogenicity and increases the risk of pregnancy complications and malformations (35–37). Therefore, amantadine should be avoided during pregnancy. Other anti-PD medications, including anticholinergics, catechol-O-methyltransferase (COMT) inhibitors, and monoamine oxidase-B (MAO-B) inhibitors, are rarely used as monotherapy or alone with levodopa or combined with dopamine decarboxylase inhibitors during pregnancy, and adverse effects on fetal development have been reported.

## Conclusions

We reported the case of a Chinese woman who was diagnosed with YOPD, with homozygous *PRKN* mutation, and who received levodopa/benserazide treatment during pregnancy. Although the patient stopped levodopa/benserazide due to an improvement in PD symptoms during pregnancy, we believe that levodopa/benserazide is safe for the treatment of patients with *PRKN*-related PD during pregnancy. More data on the safety of anti-PD drugs used in PD treatment and the impact of pregnancy on parkinsonian symptoms are needed. Obstetricians and neurologists need to learn how to manage pregnancy in patients with PD to ensure optimal maternal and infant outcomes.

## Data availability statement

The original contributions presented in the study are included in the article/supplementary material, further inquiries can be directed to the corresponding author.

## Ethics statement

Ethical review and approval was not required for the study on human participants in accordance with the local legislation and institutional requirements. The patients/participants provided their written informed consent to participate in this study. Written informed consent was obtained from the individual(s) for the publication of any potentially identifiable images or data included in this article.

## Author contributions

H-xL was responsible for drafting and revision of the manuscript. MD and KZ were responsible for the revision of the manuscript. X-xP, Y-zL, HW, CL, and Y-yD were responsible for collecting the data. H-xL and QZ were responsible for the concept and revision of the manuscript. All authors contributed to the article and approved the submitted version.
